# Dysregulation of Central–Medial Amygdala Histone Modifiers in Preclinical Models of Ethanol Exposure

**DOI:** 10.1111/adb.70095

**Published:** 2025-11-05

**Authors:** Kara A. Lamb, Alexander V. Margetts, Claude‐Henry Volmar, Florence Bourgain‐Guglielmetti, Claes Wahlestedt, Luis M. Tuesta

**Affiliations:** ^1^ Center for Therapeutic Innovation University of Miami Miller School of Medicine Miami Florida USA; ^2^ Department of Psychiatry and Behavioral Sciences University of Miami Miller School of Medicine Miami Florida USA; ^3^ Sylvester Comprehensive Cancer Center University of Miami Miller School of Medicine Miami Florida USA

**Keywords:** alcohol use disorder (AUD), central–medial amygdala, epigenetics, ethanol‐exposure, histone modifiers

## Abstract

This narrative review examines recent advances in understanding the epigenetic and transcriptional dynamics of the central–medial amygdala across different stages of ethanol use. It covers the various models and protocols of ethanol administration, emphasizing their strengths and limitations in helping understand ethanol‐induced changes in brain and behaviour. The findings from protocols utilizing acute, chronic‐intermittent ethanol (CIE) and chronic‐continuous ethanol (CCE) exposure are summarized into a proposed mechanism of epigenetic dysregulation and neuroadaptation, spanning from initial ethanol exposure to withdrawal and its influence on gene expression and neuronal activity. This review also explores potential epigenetic targets for therapeutic intervention. Understanding the mechanisms that underlie histone remodelling during initial ethanol exposure, chronic exposure, and withdrawal has the potential to elucidate novel cessation strategies tailored to specific stages of alcohol use disorder (AUD).

## Graphical abstract

This narrative review examines the epigenetic and transcriptional dynamics of the central–medial amygdala across stages of ethanol use. It outlines acute, chronic intermittent, and chronic continuous ethanol exposure models, highlighting their strengths and limitations for understanding ethanol‐induced changes in brain and behaviour. The findings from protocols utilizing these models are summarized into a proposed mechanism of epigenetic dysregulation and neuroadaptation from initial exposure to withdrawal. The review also explores epigenetic targets for therapeutic intervention in alcohol use disorder.

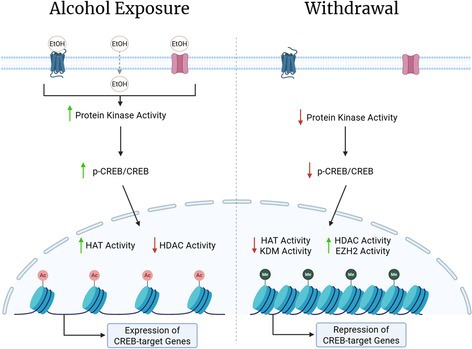

## 1 Introduction

Alcohol use disorder (AUD) is a chronic substance use disorder characterized by compulsive seeking and prioritization to consume alcohol despite negative consequences. A survey of the prevalence of AUD in the United States of America in people aged 12 years and older found that 28.9 million individuals (10.2% in the age group) reported AUD in the past year [1]. Despite being a disorder with high genetic heritability, environmental factors that affect genetics can also play an important role in influencing the risk of developing AUD [2]. To this end, recent efforts into the mechanistic links between environmental factors and gene expression changes that contribute to the development and progression of AUD [3] have implicated a role for epigenetic regulation. Specifically, erasure or deposition of histone post‐translational modifications (hPTMs) can remodel chromatin, leading to transcriptionally permissive or repressive regions, respectively [4]. Indeed, increasing evidence suggests that epigenetic neuroadaptations associated with habitual ethanol exposure may influence the course of AUD [5].

Chromatin is organized into nucleosomes composed of 147 DNA base pairs that loop around an octamer of core histone proteins (H2A, H2B, H3 and H4). Histone proteins undergo post‐translational modifications at the N‐terminal amino acid residues that alter chromatin accessibility [6, 7]. Condensed (closed) chromatin is associated with transcriptional repression, whereas accessible (open) chromatin is conducive to transcriptional activation [4]. The combination of modifications on histones, along with their varying degrees, that results in the precise control of chromatin compaction is referred to as the histone code [8]. Various types of hPTMs exist, including, but not limited to: acetylation, methylation, phosphorylation and ubiquitylation [6]. Several studies have focused specifically on the histone acetylation and methylation patterns in the central–medial amygdala of compulsive alcohol models.

The central–medial amygdalar complex, including the central amygdala (CeA) and medial amygdala (MeA), serves as a critical hub for the interactions of neuromodulators that mediate fear and anxiety behaviours and are associated with alcohol withdrawal [9], with each nucleus engaging in distinct aspects of this process. The CeA and MeA interact anatomically and functionally; the CeA primarily regulates emotional valence and responses to fear and stress, while the MeA integrates input from various brain systems to support social behaviours [9]. Alcohol‐induced neuroadaptations alter neuropeptide signalling—key mediators of stress and reward pathways—contributing to the dysregulation of brain stress systems and other maladaptations [10, 11, 12]. The ethanol‐induced neuroadaptive mechanisms produce emotional stress as the amygdala projects largely to effector regions such as the hypothalamus and brainstem [13]. These connections drive behaviours related to fear and anxiety that serve as negative reinforcement, promoting relapse and alcohol dependence [13].

In this review, we examine experimental protocols for studying ethanol‐induced alterations in the central–medial amygdala, highlighting key findings of hPTM patterns across different models of ethanol exposure and their interconnected mechanisms, specifically focusing on hPTMs. We also discuss potential therapeutic strategies targeting epigenetic modifying enzymes or genes under epigenetic control that are differentially expressed following ethanol‐induced dysregulation at various stages of AUD.

To identify relevant literature, searches were conducted using the USearch platform (University of Miami's discovery system) and PubMed for articles published between 2010 and 2025. Various combinations of the following keywords were used: *amygdala, alcohol, ethanol, histone, histone modification, epigenetic, withdrawal* and *alcohol use disorder*. Studies that investigated epigenetic regulation in the context of alcohol exposure with a specific focus on amygdalar subregions were included. Studies discussing the amygdala without distinguishing between its nuclei were excluded, as the review focuses on mechanisms specific to the central–medial amygdala. Given the broad scope of the addiction field and the numerous brain regions implicated, these criteria were necessary to refine the scope and maintain a clear focus.

## 2 Models of Alcohol Administration

A variety of experimental designs and ethanol administration protocols are utilized in the study of alcohol use and dependence, each optimized to model specific behaviours and underlying neurobiological mechanisms. Here, we will focus on models that have contributed to the understanding of ethanol‐induced modulation and neuroadaptation in the rat amygdala. These models are broadly categorized into (1) acute ethanol exposure, (2) chronic intermittent ethanol (CIE) exposure and (3) chronic continuous ethanol (CCE) exposure, each of which is summarized in Table 1.

**TABLE 1:** Ethanol exposure models and administration protocols.

Exposure type	Description	Administration protocol
Acute ethanol exposure	Model for short‐term impacts of alcohol on brain and behaviour	Intragastric (IG): Oral gavage (e.g., 3× daily) [14]
		Intraperitoneal (IP) injection: Single doses [15, 16, 17]
Chronic intermittent ethanol (CIE) exposure	Mimics human binge drinking patterns with exposure and withdrawal cycles	Intragastric: Oral gavage over extended period [14]
		Intraperitoneal injection: AIE (8 doses over 2 weeks) [16, 18]
		Alcohol vapour: Precise and prolonged vapour exposure [19, 20]
		Intermittent alcohol self‐administration: Voluntary intermittent access to ethanol intake via completion of tasks [10, 20, 21]
Chronic continuous ethanol (CCE) exposure	Prolonged, uninterrupted alcohol intake for long‐term effects	Intraperitoneal: RET (back‐to‐back doses) [15]
		Continuous alcohol self‐administration: Voluntary unlimited access to ethanol intake via completion of tasks [20, 22, 23]

*Note:* Overview of ethanol exposure models, their purpose and key administration methods.

### 2.1 Acute Ethanol Exposure

Acute ethanol exposure is a valuable model for investigating the immediate effects of ethanol on brain function and behaviour. This model enables researchers to study the short‐term effects of alcohol consumption, offering insights into the initial stages of tolerance development and the mechanisms driving the rewarding effects of ethanol [15]. Acute ethanol exposure protocols discussed in this review include intragastric (IG) administration of ethanol via oral gavage and intraperitoneal (IP) injection of ethanol [14, 16, 17]. IG administration of ethanol three times daily has been shown to result in consistent blood alcohol concentrations and is designed to mimic acute binge drinking [24]; however, as an involuntary administration method, gavage may induce stress, potentially confounding behavioural and molecular results [24]. IP injection is used for precise single doses of ethanol, ensuring rapid absorption and controlled experimental conditions to model acute intoxication in humans [25]. However, the clinical relevance of IP ethanol administration may be limited because humans do not typically consume alcohol through injection, and IP ethanol injections may cause pain, exacerbate local inflammation and trigger stress responses, all of which may confound immune and behavioural assessments [24, 25].

### 2.2 Chronic Intermittent Ethanol Exposure

CIE exposure simulates human binge drinking patterns that are characterized by repeated cycles of alcohol exposure and withdrawal, and subsequent dependence on alcohol. This model is useful for identifying neuroadaptations that develop over time, even in the absence of alcohol during withdrawal periods [14, 21, 22]. CIE protocols covered in this review include IG, adolescent intermittent ethanol (AIE), alcohol vapour exposure and intermittent alcohol self‐administration (ASA) access. IG administration, while similar to acute ethanol exposure protocols, occurs over several consecutive days, such as three times daily (total daily dose of 4.5 g/kg) for 5 days [16, 18]. AIE administration is conducted by 8 IP injections of ethanol (2 g/kg) over 2 weeks (2‐d on/off) to model the impact of ethanol exposure on the developing adolescent brain; this protocol mimics human cycles of binge drinking followed by abstinence to study the long‐term effects of adolescent drinking [16, 18]. Alcohol vapour exposure induces dependence by precise exposure to ethanol vapour for prolonged periods followed by periods of abstinence [19, 20]. A benefit of alcohol vapour exposure is the ability to reach clinically relevant blood alcohol concentrations (BAC) to mimic chronic heavy drinking (BACs between approximately 200 and 300 mg/dL) [19, 20]. Intermittent ASA access is a voluntary ethanol administration protocol where animals are trained to perform operant tasks to self‐administer low ethanol concentrations (typically 3%–20% v/v) during intermittent access periods. Following acclimatization, recent studies have increased concentrations to as high as 60%, enhancing its clinical relevance for modelling the binge‐intoxication stage of AUD [10, 18, 21, 22].

### 2.3 Chronic Continuous Ethanol Exposure

CCE exposure involves prolonged, uninterrupted alcohol intake to understand the long‐term effects on the brain and the severe withdrawal syndrome associated with AUD [20, 23, 26]. CCE models are often adapted from previously described protocols for long‐term exposure. Recent studies have employed rapid ethanol tolerance (RET) and continuous ASA access to represent CCE. RET is conducted by back‐to‐back IP administrations of ethanol (1 g/kg of ethanol, 24 h apart; 2 g/kg ethanol for tolerant groups) within a short time frame, bypassing a period of abstinence [15]. RET reflects the rapid adaptation of the brain to the effects of ethanol and is a useful protocol for exploring tolerance in humans. Continuous ASA access mirrors intermittent ASA access, with the key difference being uninterrupted access to ethanol. This protocol is useful for representing human drinking behaviour in environments with unlimited alcohol availability. However, it may lead to lower ethanol consumption compared to intermittent access protocols, as animals may be less motivated to maximize intoxication with unlimited alcohol access [20, 21].

### 2.4 Involuntary and Voluntary Ethanol Administration

Ethanol administration methods can be further subdivided into involuntary and voluntary administration. Common protocols of involuntary ethanol administration are IG, IP and alcohol vapour exposure [14, 15, 18, 20]. These protocols offer precise control over ethanol dosage and timing, making them easier to reproduce and scale across studies compared to voluntary administration. The consistent findings from studies using these protocols have been instrumental in building foundational knowledge about the hPTMs associated with various drinking patterns [27]. However, involuntary administration models do not incorporate the motivational and decision‐making aspects of AUD that contribute to dependency, thus limiting their translational potential. Additionally, some involuntary methods produce blood alcohol concentration levels that far exceed those typically seen with human intoxication [22, 27].

Voluntary ethanol administration typically involves training animals to perform a task resulting in the delivery of ethanol [23, 28]. Voluntary administration is useful for studying the motivational aspects of ethanol‐taking, measuring withdrawal behaviour, and evaluating pharmacological interventions that may influence volitional ethanol consumption. However, voluntary ethanol administration introduces several important variables that are absent in noncontingent, or involuntary, models: Training for self‐administration can vary widely across subjects; environmental stressors and ethanol preference can influence self‐administration rates and affect the pharmacokinetic and pharmacodynamic profile in each subject [29, 30]. Recent studies have used different protocols and routes of ethanol administration to uncover specific regional and temporal epigenetic and transcriptional changes in the central–medial amygdala. Many of these studies also examined the resulting withdrawal states (Table 1).

## 3 Histone Post‐Translational Modification Patterns in the Central–Medial Amygdala Associated With Compulsive Ethanol Consumption

### 3.1 Histone Acetylation Promotes an Open Chromatin State

Histone acetylation is a key regulatory mechanism that dynamically alters gene expression, notably in response to ethanol exposure in the amygdala [31]. This hPTM involves the addition of an acetyl group to a lysine residue, neutralizing its positive charge, weakening histone–‐DNA interactions and promoting active transcription [7, 8]. The acetyl group is written by histone acetyltransferases (HATs) and removed by histone deacetylases (HDACs) [7]. HATs catalyze the acetyl group transfer from acetyl‐CoA to the ε‐amino group of lysine side chains, modifying multiple sites on the N‐terminal histone tail and within the globular histone core through interactions with various transcriptional coactivators and larger protein complexes [8]. HDACs counteract the effects of HATs by removing acetyl marks, thus restoring the positive charge of the lysine and strengthening the DNA‐histone interaction, leading to a condensed chromatin structure associated with transcriptional silencing [7]. HDAC recruitment and specificity are ambiguous because of their relatively low substrate specificity, enabling the modification of multiple sites within histones; these are also typically present within distinct complexes—often with other HDAC proteins [8]. Ethanol exposure produces hPTM patterns that are unique across acute exposure, chronic exposure and withdrawal stages of alcohol use.

The anxiolytic and euphoric effects associated with acute ethanol exposure are associated with specific epigenetic modifications and transcriptional changes in amygdalar regions. A study that used a combination of qPCR, gold immunolabelling and colorimetric activity assays indicated that acute ethanol exposure reduced HDAC activity and increased histone 3 (H3) and histone 4 (H4) acetylation in amygdalar brain regions of rats [31]. In the CeA and MeA, the activated phosphorylated form of cAMP‐responsive element binding (p‐CREB), CREB‐binding protein (CBP) and neuropeptide Y (NPY) protein levels were increased. CBP has intrinsic HAT activity, which may account for the increased histone acetylation seen in the CeA and MeA [31]. Another study using assay for transposase‐accessible chromatin using sequencing (ATAC‐seq), RNA sequencing (RNA‐seq), integrative data analysis, chromatin immunoprecipitation‐qPCR (ChIP‐qPCR), and qPCR found that acute ethanol exposure increased H3 lysine (K) 27 acetylation (ac), H3K9ac and H3K14ac at the promoter region of hypoxia‐inducible factor 3 alpha subunit *(Hif3a*), resulting in its increased expression and activity in the CeA [17]. HIF3A functions as a transcription factor and has a role in several regulatory pathways. Indeed, HIF3A has been shown to occupy promoter regions of *Npy* and activity‐regulated cytoskeleton‐associated protein (*Arc)*, leading to their increased expression. The knockdown of *Hif3a* in the CeA attenuated ethanol‐induced *Hif3a* mRNA expression and blocked anxiolysis in rats, implicating its role in the transcriptional changes induced by ethanol exposure [17].

Transcriptional changes induced by acute ethanol exposure were found to normalize with repeated exposure. In the rat amygdala following chronic ethanol exposure, the decreased HDAC activity, increased H3K9ac and H3K8ac levels and elevated CBP expression observed during acute ethanol exposure were normalized [17]. Another study used RET to induce compulsive behaviour in rats, re‐establishing the anxiolytic effects experienced during initial ethanol exposure [15]. A single ethanol exposure (1 g/kg) reduced HDAC activity and increased histone acetylation and *Npy* mRNA expression in the CeA and MeA; however, two ethanol exposures administered 24 h apart failed to elicit a response in these regions until the dosage was increased (2 g/kg) [15]. Following the dosage increase, amygdalar HDAC activity and anxiolysis comparable to the initial ethanol exposure were observed. These findings were supported by qPCR, gold immunolabelling and colorimetric activity assays. Notably, the majority of these changes were co‐localized with neuronal nuclei (NeuN)‐positive neurons in amygdalar structures, as shown by confocal imaging, suggesting the neuronal populations in the amygdala contribute to the progression of RET [15]. A separate study employed an alternative model that may drive ethanol‐induced neuroadaptation, AIE exposure, to investigate its effects on neuromodulators and their regulation by histone acetylation in adulthood. In the CeA and MeA of AIE‐exposed adult rats, alpha‐melanocyte stimulating hormone (α‐MSH) and melanocortin 4 receptor (MC4R) were increased, while NPY protein expression was decreased. In support of these findings, H3K9ac and H3K14ac levels were decreased at the *Npy* promoter but increased at the *Mc4r* promoter in the amygdala of AIE‐exposed adult rats [18]. These findings, supported by qPCR, gold immunolabelling and ChIP‐qPCR, identified aberrant histone acetylation patterns leading to the dysregulation of the melanocortin system and reduced NPY expression, both associated with alcohol dependence, in adulthood following AIE [18, 32].

### 3.2 Histone Methylation Promotes a Condensed Chromatin State

Histone methylation involves the transfer of a methyl group to the side chains of either lysine or arginine amino acid residues on the N‐terminal histone tail, and unlike acetylation, it does not alter the histone protein's charge [8]. Lysine may be mono‐, di‐ or tri‐methylated, whereas arginine may be mono‐methylated or di‐methylated in either a symmetric or asymmetric manner [8]. Histone methylation is catalyzed by histone methyltransferases (HMTs), such as enhancer of zeste homologue 2 (EZH2); these marks can then be erased by histone demethylases (HDM), such as lysine demethylases (KDM) [7, 8]. While there are fewer studies covering amygdalar histone methylation patterns in alcohol compulsive models than histone acetylation, persistent alterations in histone methylation have been implicated in neuroplastic and neuroimmune changes within the reward system [19]. Pandey and collaborators have spearheaded this effort with several studies utilizing the AIE model.

The adult AIE rat amygdala exhibited numerous changes in histone modifier expression and associated modifications, as demonstrated by qPCR, ChIP‐qPCR, gold immunolabelling and confocal immunofluorescence (IF). Lysine demethylase 6b (KDM6B) and CBP levels, along with H3K27ac occupancy at the *Arc* synaptic activity response element (SARE) site, were decreased, while H3K27 tri‐methylation (me3) occupancy at the *Arc* SARE site increased [33]. This study also employed the targeted knockdown of *Kdm6b* and *Arc* eRNA in the CeA of control rats via small interfering (si)RNA infusion, which induced anxiety‐like behaviours and mimicked the histone modifier and modification changes observed after AIE. A recent study built on these findings with CRISPR‐based targeted epigenetic editing using dCas9 fused to either P300, a histone acetyltransferase, or Krüpell‐associated box (KRAB), a transcriptionally repressive domain, along with *Arc* SARE single‐guide RNAs (sgRNAs). They found that *Arc* SARE can bidirectionally modulate behavioural changes induced by adolescent alcohol exposure in rats, largely through epigenetic remodelling at the *Arc* promoter: P300 promoted occupancy of the transcriptionally permissive mark, H3K27ac, whereas KRAB promoted occupancy of the repressive mark, H3K27me3 [34]. KDM6B is reported to form complexes with CREB pathway proteins, such as CBP, to regulate the expression of neuronal activity‐regulated genes [33]. The AIE‐induced occupation of H3K9 di‐methylation (me2) at the brain‐derived neurotrophic factor (*Bdnf)* promoter is linked to its decreased acetylation, whereas acute ethanol exposure produces the opposite effect, increasing histone acetylation at the *Bdnf* promoter, as shown by ChIP‐qPCR [35].

A study that utilized qPCR, ChIP‐qPCR and IF found that the amygdala of adult AIE rats showed a similar epigenetic profile to that of the human postmortem amygdala of individuals diagnosed with AUD [36]. This profile included increased EZH2 protein levels, increased repressive H3K27me3, decreased H3K27ac occupancy at the *Arc* SARE site and decreased ARC protein expression. In the AIE model, EZH2 protein levels were significantly higher in a subpopulation of GABAergic neurons in the CeA, specifically those expressing protein kinase C‐delta (PKCδ). The knockdown of *Ezh2* via siRNA infusion into the CeA prevented AIE‐induced anxiety‐like behaviour, normalized H3K27me3 and H3K27ac levels at the *Arc* SARE site and restored ARC expression. Additionally, increasing H3K27me3 by knocking down *Kdm6b* in the CeA of adult ethanol‐naïve rats induced an anxiety‐like phenotype resembling that of adult AIE rats [36]. Collectively, these findings suggest a causal link between EZH2‐mediated epigenetic changes and the anxiety‐like phenotype associated with AUD.

In adult AIE rats, lysine‐specific demethylase 1 (*Lsd1*) and *Kdm6b* expression were decreased, concordant with an increase in H3K9me2, a generally repressive hPTM, levels in the CeA. H3K9me2 levels were specifically increased at CREB‐target genes, such as *Bdnf* and *Arc*, as shown by qPCR and ChIP‐qPCR [16]. A similar study noted that decreased expression of *Lsd1* was localized to the CeA, but not the MeA, where H3K9me2 levels increased without a corresponding change in H3K4me2. Additionally, *Kdm4c*, a histone demethylase that can form a complex with *Lsd1* to promote H3K9 demethylation, also showed decreased expression. HDAC2 protein expression was increased, supporting its role in the erasure of acetyl marks to allow methylation of those residues, and promoting the transcriptional repression of genes within the associated condensed regions, as demonstrated through ChIP‐qPCR, qPCR, gold immunolabelling and IF [35]. Furthermore, a separate study found that the knockdown of Hdac2 via siRNA infusion into the CeA of selectively bred alcohol‐preferring rats attenuated anxiety‐like behaviours and voluntary alcohol consumption, while increasing expression and promoter acetylation of *Bdnf* and *Arc*, as well as increasing dendritic spine density [37]. These alterations highlight the complex and selective nature of alcohol‐induced epigenetic changes.

In a study to determine the role of the endogenous opioid system in susceptibility to alcohol dependence, IG administration of ethanol and ChIP‐qPCR were utilized in rats [14]. Ethanol exposure (1.5 g/kg in 1.5 h intervals for a total daily dose of 4.5 g/kg) was found to selectively alter opioid gene expression in the amygdala. After 1 day of alcohol administration, an inverse relationship was noted between H3K27me3 and H3K9ac in the amygdala: H3K27me3 was decreased while H3K9ac was increased [14]. These findings indicate that ethanol exposure shifts the balance of hPTMs in the amygdala, influencing gene expression.

Rapid ethanol tolerance was shown to result in decreased HDAC activity and increased HAT activity following the initial acute ethanol exposure in the amygdala of rats [15]. A similar study using the RET model found that acute ethanol exposure (1 g/kg) reduced G9a histone methyltransferase and H3K9me2 levels while increasing NPY expression in the CeA and MeA. Additionally, a tolerance group received a second ethanol dose 24 h later that resulted in no significant changes to either G9a or H3K9me2 levels until the dosage increased to 2 g/kg. These findings, supported by ChIP‐qPCR and gold immunolabelling, indicated that ethanol exposure may regulate the expression of these modifiers [38]. This evidence highlights the adaptive nature of histone modifiers in response to ethanol exposure, suggesting a finely tuned regulatory mechanism to maintain homeostasis.

## 4 Proposed Model of CREB‐Mediated Transcriptional Regulation in Acute Ethanol Exposure and Withdrawal

Acute ethanol exposure leads to an increase of p‐CREB by activation of a range of protein kinases. Activated p‐CREB recruits CBP, which forms a complex with its transcriptional coactivator, p300. CBP/p300 exhibits histone acetyltransferase activity, increasing H3 and H4 acetylation and facilitating the recruitment of transcription factors at CREB‐target genes to promote their expression, including *Bdnf*, *Npy* and *Arc*, while concurrently reducing histone deacetylation [27]. Through CREB activation, BDNF and ARC have roles in learning, memory, synaptic plasticity and neuroprotection [31]. NPY is a potent anxiolytic that may suppress corticotropin‐releasing factor (CRF) and modulate GABAergic pathways in the CeA [17, 31]. While other transcription factors contribute to alcohol‐related transcriptional regulation, this review focuses on CREB as a key upstream regulator that affects histone modifier activity.

In contrast, CREB activation in the CeA decreases during ethanol withdrawal without changes in CREB expression, reflecting the reduced protein kinase activity [39]. With decreased p‐CREB, the expression of downstream histone modifiers is altered: the expression of HMTs and HDACs increased while HATs and HDMs expression decreased [31]. With this change in histone modifiers, the levels of H3K27me3 and H3K9me2 increased while H3K27ac, H3K9ac and H3K14ac decreased. The increased methylated histone state leads to chromatin compaction at regions of CREB‐target genes, producing an expression pattern opposite to that observed with acute ethanol exposure [14, 26]. The neuroadaptation within the central–medial amygdala is the result of repeated ethanol exposure and leads to a reliance on ethanol as an external stimulus. In the state of withdrawal, the absence of ethanol results in subthreshold stimulation and dysregulation of neuropeptide systems and signalling in the amygdala [36]. Several transcriptional and epigenetic changes previously described in acute ethanol exposure tend to reverse in withdrawal [31].

**FIGURE 1:**
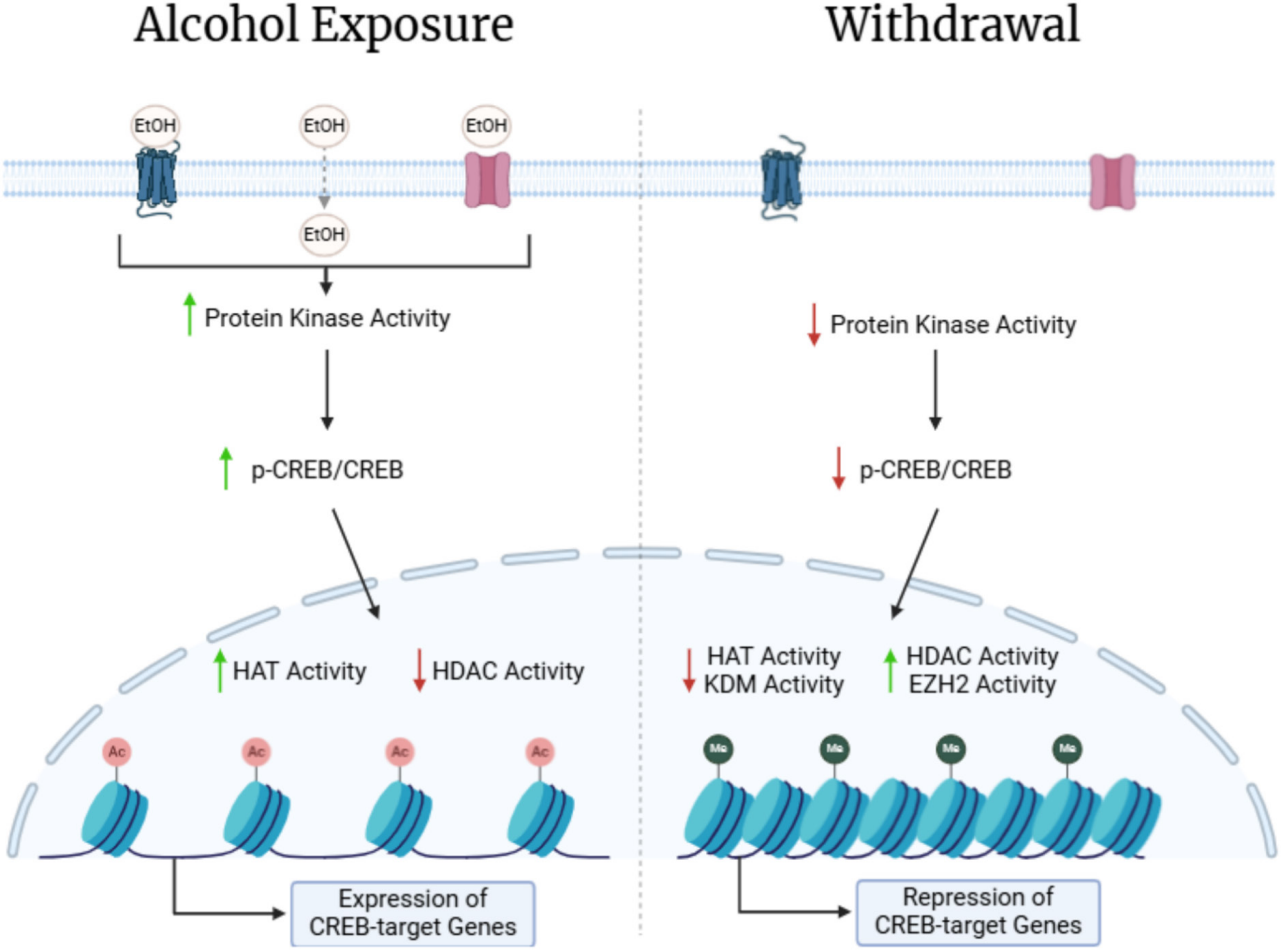
Model of ethanol‐induced epigenetic modulation and chromatin remodelling during alcohol exposure and withdrawal. Ethanol accumulates in the extracellular matrix following alcohol exposure. Through various interactions, ethanol exposure leads to increased protein kinase activity, phosphorylation of CREB, increased HAT and decreased HDAC activity that promotes an open chromatin state, allowing the expression of CREB‐target genes. Following neuroadaptive changes within the cell and loss of ethanol to stimulate the cell, withdrawal results in reduced protein kinase activity, decreased phosphorylation of CREB, decreased HAT and KDM activity and increased HDAC and EZH2 activity that results in a compacted chromatin state, repressing the transcription of CREB‐target genes. Created in BioRender [64].

As summarized by the model presented in Figure 1, cellular responses to acute ethanol exposure and subsequent withdrawal reflect inverted patterns of the same signalling pathways, resulting from three distinct molecular shifts: First, kinase activation of CREB is an upstream driver of the histone and transcriptional changes. Following acute ethanol exposure, CREB phosphorylation increases, thereby activating the downstream signalling cascade that drives the expression of CREB‐target genes. In a withdrawal state, CREB phosphorylation is reduced without a reduction in CREB expression, suggesting a reduction in upstream kinase activation [39, 40]. Second, the activity and expression of histone modifiers are distinct between acute ethanol exposure and withdrawal. Acute ethanol exposure induces histone acetylation and reduces histone methylation to promote the transcription of CREB‐target genes by producing an open‐chromatin state [38]. However, this hPTM pattern is reversed during withdrawal, as permissive histone acetylation marks are replaced with repressive methylation marks, producing a compacted chromatin state [40]. Third, this compacted chromatin state results in differential expression of CREB‐target genes, altering neuronal activity and signalling between acute ethanol exposure and withdrawal [31].

## 5 Targeting Histone Modifiers in AUD

While most available treatments for addiction act on the initial protein targets for drugs of abuse, the molecular dynamics of histone modifiers in the central–medial amygdala offer a range of potential druggable targets for the treatment of AUD [41]. The distinct hPTM patterns observed in the acute, chronic and withdrawal states of ethanol exposure suggest that targeted interventions towards specific histone modifiers could be tailored to treat patients at each stage of AUD. Histone modifiers present a promising avenue of drug intervention to reprogram AUD‐associated gene expression dysregulation in the central–medial amygdala.

The acute and chronic states of ethanol exposure are associated with upregulated HATs and HDMs that remove repressive methylation marks and produce an open‐chromatin state at CREB‐target gene regions through the deposition of histone acetylation marks [31]. Therefore, the inhibition of these histone modifiers during the acute and chronic states of ethanol exposure may restore normal hPTM patterns. However, HATs have proven to be difficult drug targets due to their functional promiscuity; Type‐A HATs, such as the CBP/p300 families, can acetylate multiple sites on a histone tail and core through their interactions with large multiprotein complexes and transcriptional coactivators, such as HDMs [8, 42].

While both HATs and HDMs are druggable, the inhibition of HDMs may be a preferred option. As mentioned previously, HDMs and HATs work synergistically to promote an open‐chromatin state; HDMs can remove methyl marks from histone lysine residues, allowing HATs to acetylate the residue. Inhibiting HDMs may reduce the ability of HATs to acetylate specific residues without affecting their other functions and targets. Given that HDMs have a higher level of target specificity than HATs [8], inhibiting HDMs may prove more specific to correcting aberrant histone acetylation at CREB‐target regions [7, 8].

The withdrawal state following ethanol‐induced neuroadaptation is characterized by a global increase in histone methylation in the central–medial amygdala. The histone modifiers that are prevalent in this state are HMTs and HDACs [8]. HMTs may display distinct substrate specificity and are not as dynamic as HATs. Histone methylation is often associated with long‐term gene silencing that implies a level of stability to the mark [43]. Additionally, HMTs demonstrate varying baseline expression patterns in amygdalar subregions: in the CeA and MeA, but not the basolateral amygdala, HMT expression and associated methylation are selectively increased [40]. The specificity of HMTs and the stability of their repressive methylation marks may make these a compelling target for therapeutic intervention.

HDACs are upregulated in the central–medial amygdala during withdrawal [35]. Through its increased activity, the catalyzed sites become available for other hPTMs, particularly repressive methylation modifications, which may promote a closed‐chromatin state at the regions of CREB target genes [16]. However, like HATs, HDACs have low substrate specificity and target precise gene regions through the function of multiprotein complexes [7]. Thus, the inhibition of HDACs may also produce numerous off‐target effects. A better strategy may be to identify a druggable target that specifically and directly regulates HDAC recruitment to certain sites. The synergistic relationship between HMTs and HDACs suggests there may be shared regulatory mechanisms; therefore, the inhibition of HMTs may indirectly modulate HDAC activity and remain a promising target.

While epigenetic drugs hold significant promise in modifying the chromatin landscape to restore proper gene expression, their clinical use in humans remains limited [44]. Most investigations involving these compounds focus on cancer, where the primary goal is to selectively target cancer cells—particularly those dependent on epigenetic mechanisms for survival [45]. Dosing strategies vary depending on the indication: higher doses are typically used in cancer settings to induce cytotoxicity, whereas lower doses may be more appropriate for non‐cancer conditions, such as AUD, where the objective is to restore gene expression without toxicity. Many studies also report the strong potential of combining epigenetic drugs with other therapies to achieve synergistic effects, further limiting the toxicity and broad off‐target actions often associated with epigenetic drugs [45].

## 6 Future Directions: Studying the Epigenetics of AUD

The study of epigenetic mechanisms in AUD has been met with several challenges, primarily arising from the cellular heterogeneity of the brain regions involved [46]. Neural cell types can exhibit differential responses to ethanol exposure, which may confound bulk transcriptional and epigenetic profiling analyses [47, 48]. Traditional methods that analyse bulk tissue samples often capture an average response from all cell types, potentially masking the changes occurring within specific cell types [49, 50]. This presents a significant barrier to understanding how gene expression dysregulation in different cell types contributes to AUD.

However, more recent molecular profiling techniques have enabled the analysis of gene expression and epigenetic changes at both single‐cell and cell‐type resolution [51, 52, 53, 54, 55]. Building on bulk RNA‐seq, single‐cell RNA‐seq (scRNA‐seq) involves the sequencing of genes expressed within individual cells following single‐cell isolation [56]; as a result, rare neuronal or glial populations that were masked in bulk assays have been distinguished [57, 58]. In parallel, chromatin‐profiling methods have evolved beyond ChIP‐seq, which required around 1 million cells as input [59]. Cleavage Under Targets and Release Using Nucleases (CUT&RUN), Cleavage Under Targets and Tagmentation (CUT&Tag) and Dynamic targets and Tagmentation (DynaTag) are low‐input chromatin profiling techniques that make use of specific antibodies tethered to enzymes to generate high signal‐to‐noise sequencing libraries for profiling transcription factors, hPTMs and other chromatin components [53, 60, 61]. CUT&RUN, CUT&Tag and DynaTag overcome previous input limitations by producing amplified sequencing libraries with minimal background—even when starting with as few as 500 cells per chromatin modifying target [54, 55], as is often necessary when studying discrete cell populations and subpopulations in the brain [53, 54, 55, 62].

Furthermore, combining these approaches with emerging spatial profiling techniques provides a deeper understanding of the cellular and molecular underpinnings of AUD. For example, a combination of scRNA‐seq and multiplexed error‐robust fluorescence in situ hybridization (MERFISH) was used to generate a transcriptional and spatial profile of the nucleus accumbens, a small but functionally critical region in the brain's reward system, at single‐cell resolution [62]. Although Cleavage Under Targets‐based spatial approaches have been described and hold promise [63], there is currently no published evidence that these have been applied to brain profiling in addiction research, most likely due to the recency of their development.

These technologies, together, enable the simultaneous assessment of gene expression, chromatin dynamics and protein localization across brain regions and cell types, supporting the investigation of how epigenetic mechanisms shape cellular phenotypes in addiction. By analyzing regulatory networks at the cellular and spatial level, these emerging tools offer new opportunities to profile the transcriptional and epigenetic signatures of discrete cell populations and subpopulations—clarifying how gene expression dysregulation is orchestrated across AUD.

## 7 Conclusions

AUD is a multifaceted substance use disorder, driven by a complex interplay of various genetic, environmental and neurobiological factors. The interaction between the environmental factors and gene expression changes has become a key focus in addiction research. In this narrative review, we explored the neuroadaptations in the central–medial amygdala, a critical component of the brain‐reward system responsible for integrating information from multiple brain regions, in response to ethanol exposure. In the central–medial amygdala, acute ethanol exposure induces open‐chromatin states, leading to the transcription of neuromodulators that produce an anxiolytic effect. In contrast, chronic ethanol exposure normalizes these changes, and subsequent withdrawal triggers condensed chromatin states that activate stress signalling in the brain and contribute to alcohol dependence and relapse. The evidence presented here suggests that epigenetic modifying enzymes may serve as potential pharmacological targets for AUD intervention. However, given the complexity of epigenetic regulation across cell types and brain regions, leveraging novel epigenomics techniques will aid in uncovering cell‐ and phase‐specific targets for the development of novel therapeutics to treat AUD and support long‐term recovery.

## Data Availability

Data sharing is not applicable to this article as no datasets were generated or analysed during the current study.
